# Objective Food Intake in Night and Day Shift Workers: A Laboratory Study

**DOI:** 10.3390/clockssleep1010005

**Published:** 2018-10-14

**Authors:** Yichi Chen, Shaza Lauren, Bernard P. Chang, Ari Shechter

**Affiliations:** 1 Institute of Human Nutrition, Columbia University Irving Medical Center, New York, NY 10032, USA; 2 Department of Emergency Medicine, Columbia University Irving Medical Center, New York, NY 10032, USA; 3 Center for Behavioral Cardiovascular Health, Columbia University Irving Medical Center, New York, NY 10032, USA

**Keywords:** shift work, short sleep, food intake, obesity, diet, macronutrients

## Abstract

Night shift work is associated with risk of overweight and obesity. In night shift workers, short sleep duration combined with circadian misalignment may contribute to altered food intake regulation, favoring positive energy balance and weight gain. Prior work investigating food intake in shift workers has suffered methodologically due to reliance on subjective self-report for dietary assessment. No study has yet been done to examine the impact of night shift work on food intake in real-life shift workers using objective measures. Female day (*n* = 12) and night (*n* = 12) shift workers from a hospital setting participated in a laboratory-based objective food intake assessment. Participants entered the laboratory in the fasted state after awakening from the sleep episode following a final work shift, and underwent an ad libitum 14-item test meal buffet to objectively quantify food choice/intake. Sleep duration (measured via wrist-accelerometry) during the sleep episode before laboratory assessment was significantly longer in day vs. night workers (373.9 ± 127.5 vs. 260.6 ± 102.9 min, *p* = 0.03). No significant group difference was observed in calories consumed during the test meal (943.08 ± 469.55 vs. 878.58 ± 442.68 kcal, *p* = 0.74). When expressed as percent of energy consumed, day workers had higher protein consumption vs. night workers (16.03 ± 5.69 vs. 11.82 ± 4.05%; *p* = 0.05). To our knowledge, this is the first laboratory-based behavioral assessment of food choice/intake in actual night and day shift workers. Although not studied here, work by others has linked protein intake to satiety. This may be a potential pathway placing shift workers at risk for overweight and obesity.

## 1. Introduction

Obesity is a leading health concern in the United States, where the prevalence is over one in three adults in the population [1]. This problem may be exacerbated by shift work: Recent meta-analyses demonstrate that shift work [2], and in particular, night shift work [3], is associated with a significantly increased risk of overweight and obesity. This increased obesity risk is critical, since up to 29% of the U.S. workforce engages in “alternative” shift schedules, encompassing evening, night, and rotating shifts [4].

Various factors may contribute to alterations in energy balance regulation and subsequent weight gain within night shift workers. Chief among these is short sleep duration. Based on observational studies as well as mechanistic laboratory-based experiments, short sleep duration and/or sleep restriction is found to be associated with increased food intake [5]. Short sleep duration is prevalent in shift workers [6]. The effects of short sleep duration may be exacerbated in shift workers since both the sleep-wake and feeding-fasting cycles are displaced, leading to a misalignment of circadian physiology and behavior. Together, these may lead to a dysregulation of hunger/satiety hormones. A decrease in the satiety hormone leptin is seen in night shift work simulation [7], and a decrease in leptin and an increase in the hunger hormone ghrelin are seen under conditions of experimental sleep restriction combined with circadian misalignment [8]. These observations are consistent with a hormonal profile that would be expected to induce high energy intake.

Studies comparing food intake between shift and standard day workers have not consistently found differences in total daily energy or macronutrient intake [9]. However, in all cases, food intake was assessed subjectively via Food Frequency Questionnaire (FFQ), self-reported food diary, or dietary recall. There are considerable limitations in quantifying food intake with FFQ and similar self-reports. It has been suggested that these subjective tools may lead to inaccurate conclusions and, accordingly, their use should be abandoned [10]. It is therefore important to conduct further systematic investigations of food intake in shift workers using objective measures under real-life and laboratory conditions.

To date, only one study, by Cain and colleagues, assessed food intake using objective measures in participants undergoing simulated night shift work [11]. In the counter-balanced cross-over trial, significant increases in high-fat food consumption, with no differences in total energy intake, were observed in a test meal following a night shift simulation compared to control condition [11]. To our knowledge, no study has yet been done to examine the impact of night shift work on food intake in real-life shift workers using objective measures. We therefore conducted a laboratory-based behavioral assessment of food intake in actual night and day shift workers, to test the hypothesis that compared to day work, night shift work would be associated with increased intake of energy and fat in a test meal.

## 2. Results

Day and night shift workers did not differ significantly in age (day: 32.5 ± 7.4 y, range: 24–53 y; night: 37.0 ± 7.5 y, range: 25–47 y; *p* = 0.15), body mass index (BMI; day: 28.2 ± 3.1 kg/m^2^, night: 30.6 ± 4.0 kg/m^2^, *p* = 0.11), systolic blood pressure (day: 114.0 ± 9.7 mmHg, night: 117.4 ± 9.9 mmHg, *p* = 0.40) or diastolic blood pressure (day: 72.0 ± 8.0 mmHg, night: 73.8 ± 5.2 mmHg, *p* = 0.53).

Mean bedtimes and wake-times were 23:26 ± 1:44 and 6:25 ± 1:29 for day workers, and were 09:52 ± 1:01 and 14:59 ± 1:52 for night workers. Total sleep time on the night preceding laboratory assessment was significantly longer in day workers compared to night workers (373.9 ± 127.5 min vs. 260.6 ± 102.9 min, *p* = 0.03). Although participants were asked to enter the laboratory 1 h after awakening, based on sleep log and actigraphy, participants actually entered the lab about 2.3 h after awakening (day: 2:17 ± 1:05 h, night: 2:16 ± 1:08 h, *p* = 0.94).

**Figure 1:**
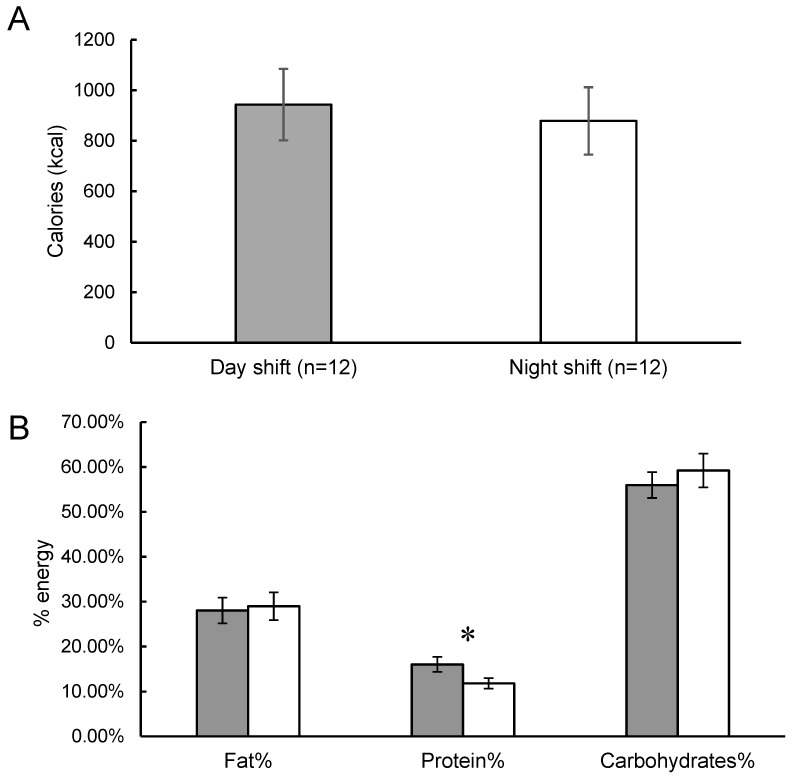
Energy and macronutrient intake from the ad libitum test meal in day (grey bars) and night (whiter bars) shift workers. Data are expressed as mean ± standard error of the mean. (**A**) Total energy intake (kcal). (**B**) Macronutrient intake expressed as a percentage of total energy consumed. * indicates *p* ≤ 0.05.

The test meal was consumed at 09:19 ± 1:29 in day workers and at 17:59 ± 1:53 in the night workers (*p* < 0.01). No significant difference between day and night shift groups was observed in total energy consumed during the ad libitum test meal (943.08 ± 469.55 kcal vs. 878.58 ± 442.68 kcal, *p* = 0.74; Figure 1A). No statistically significant differences between day and night shift groups were observed in total carbohydrates (526.33 ± 284.68 g vs. 503.00 ± 242.85 g, *p* = 0.84), fat (282.75 ± 175.88 g vs. 269.25 ± 174.19 g, *p* = 0.86), or protein (134.00 ± 50.69 g vs. 106.33 ± 56.86 g, *p* = 0.24) consumed in the test meal. When expressed as percent of energy consumed in the meal, the day shift workers had a higher protein consumption compared to the night shift workers (16.03 ± 5.69% vs. 11.82 ± 4.05%; *p* = 0.05; Figure 1B). There were no differences observed in carbohydrate (55.96 ± 9.98% vs. 59.22 ± 13.02%, *p* = 0.50) or fat (28.01 ± 9.95% vs. 28.96 ± 10.70%, *p* = 0.82) consumption in the test meal between day and night shift groups (Figure 1B). A correlation analysis between total sleep time and protein intake revealed a positive relationship that did not reach statistical significance (*r* = 0.36, *p* = 0.08).

## 3. Discussion

This study aimed to investigate the effects of night shift work on food choice and intake using objective assessment methods in actual shift workers. In a laboratory-based ad libitum test meal paradigm, we observed no difference in total energy intake between groups but that night shift workers consumed significantly less protein than day shift workers. As expected, we also observed that night shift workers had significantly shorter sleep duration than their day working counterparts.

Most observational studies in shift workers which used self-report measures of food intake do not show consistent effects of shift work type on energy or macronutrient intakes [9,12]. However, focusing on studies showing an effect on protein, an observational study using 7-day food diaries found increased protein consumption (as a percent of total energy intake) in night vs. day workers [13]. In contrast, a study using FFQs reported decreased protein intake (grams) in rotating shift workers compared to day workers [14]. In terms of sleep duration per se (in non-shift workers), short sleep duration was found to be associated with reduced protein intake, albeit when assessed with FFQ [15] or 24-h guided recall [16]. As described above, a limitation in these prior investigations is the subjective monitoring of food intake. Specifically, the FFQ has been criticized as an inaccurate measure, characterized by a systematic underestimation of actual energy intake [10]. Similar under-reporting of actual food intake based on 2-week self-reported food records [17] and diet-diary techniques [18] have also been reported.

The laboratory-based ad libitum food intake protocol is considered ideal to objectively and accurately evaluate eating behavior [19]. Current methods to assess eating behaviors under free-living conditions are seen as less valid and reliable than the controlled conditions afforded by the laboratory [20]. It is true that this laboratory-based approach sacrifices some degree of external validity and ecological relevance of a naturalistic, free-living study, albeit in favor of precision, accuracy, and internal validity. However, the strictly controlled conditions of the laboratory-based test meal are considered the ideal approach for identifying the effects of different conditions (in this case, shift type) on food intake, and are considered a good approximation of free-living conditions [20,21].

Although already commonly used to investigate the effects of experimental sleep restriction on food intake [22,23,24,25,26,27], to our knowledge, the current study is the first laboratory-based behavioral assessment of food intake in actual night and day shift workers. A prior study by Cain et al. utilized a similar ad libitum buffet approach to compare food intake under simulated night shift and control conditions [11]. In that study, a greater amount of high-fat foods, with no differences in the amount of calories or protein, was consumed following the simulated night shift compared to the control condition [11]. Several methodological differences between the two experimental protocols may account for discrepant results. As stated, their study was conducted under simulated night shift conditions in non-shift working participants. Participants in that study all had BMI between 20–25 kg/m^2^, whereas BMI ≥ 25 kg/m^2^ was an inclusion criterion in the current study. Our study focused exclusively on females, whereas the prior study had 50% female participants. Another difference between our study and the prior work by Cain et al., which may account for the increase in high-fat food consumption observed there but not here, is the composition of the test meal. In the prior study, 8 food items presented in the test meal had a high fat content (percentage of total energy of the food item ≥42%). Our test meal had 2 items with fat content >40% and another 2 items with fat content ≥29%, which we also considered to be high-fat foods. A final important protocol difference stems from the timing of the test meal relative to the work schedule and the sleep/wake episode. Specifically, in the Cain et al. study, in both the control and night conditions, the test meal was presented at 08:00. This is important, since, in the control condition, participants were eating after awakening from the nocturnal sleep episode, whereas in the night shift condition, participants were eating after an extended period of nocturnal wakefulness, including the simulated work shift. We attempted to account for this in our design by having participants arrive at the laboratory for their test meal in the fasted state after awakening from their respective day or night sleep episode. Accordingly, in our investigation, day workers and night workers were consuming the test meal at ~09:00 and ~18:00, respectively. This large difference in time introduces a potential confound. A circadian variation in hunger, with a peak in the evening and a nadir in the night/early morning, has been reported [28,29]. However, in a forced desynchrony study, the variation in hunger ratings as a function of time since last meal was relatively larger than the variation in hunger as a function of circadian phase [28]. Furthermore, without a characterization of the (altered) circadian system in night shift workers, it is difficult to determine how the differential timing of food intake relative to the endogenous circadian variation in hunger is affecting current recorded measures.

Despite the similarity in calories consumed during the test meal, the relative reduction in protein intake in night vs. day workers in the test meal observed in our study may potentially help explain the increased body weight generally noted in shift workers. Protein is considered to be the most satiating of the macronutrients [30], and a diet characterized by high relative protein intake is associated with low hunger, low food intake, and weight loss [31]. It should be emphasized that we only assessed macronutrient intake at a single test meal, and are therefore uncertain whether this pattern of relative macronutrient intake persists over the course of the 24-h day. The alterations in protein intake in the current test meal were not associated with overall differences in energy intake. This indicates that although the effect of shift type on food choice in the laboratory test meal can be considered valid, it remains speculative as to whether such a pattern can be extrapolated to fully explain the risk of overweight and obesity in night shift workers. Future work should be done to obtain objective 24-h food intake measures in shift workers who enter the laboratory for an extended period of time, or under simulated conditions. Moreover, while not studied here, a shift in the timing of food intake, as necessarily occurs in shift workers, regardless of energy or macronutrient intake, is also related to obesity [32].

This study contained several important limitations. The sample size was small. The focus on studying exclusively women who are overweight may also limit some generalizability of findings. We chose to include only overweight women to reduce variability within the small sample, to study individuals who may be at a higher risk of developing metabolic disorders, and because there is an increased prevalence of overweight and obesity in shift workers. There was some heterogeneity in the duration of shifts. Although the proportion of individuals working 8- or 12-h shifts in the day and night groups did not differ statistically, it is possible that the shift duration also impacts food intake regulation. We did not measure the palatability and desirability of the food items before or after presentation. Inter-individual differences in food preferences could therefore affect measured intakes. The buffet test meal would also benefit from a wider range of food choices. We attempted to avoid “time sensitive” foods such as traditional breakfast and lunch/dinner items in the test meal buffet, to account for group differences in the timing of the first post-sleep meal. Another potential limitation is the request that participants enter the laboratory in the fasted state for their test meal. We asked participants to refrain from ingesting any food or liquid before lab entry, however, we were unable to objectively confirm adherence to this request. Having participants undergo their sleep episode in the laboratory would be a potential solution to this limitation.

Future work on this topic should explore how the timing of food intake relates to body weight outcomes in shift workers. Other relevant energy balance-related parameters, such as hormones regulating hunger and satiety (e.g., leptin and ghrelin), energy metabolism, and physical activity-related energy expenditure should be assessed in shift workers. Such work may provide key insight into optimizing shift/sleep scheduling in healthcare providers and other shift workers at risk for disrupted sleep.

Our current findings of altered food preference and macronutrient intake in night shift workers, combined with additional well-developed and objective assessments, will help us understand the mechanisms by which sleep-wake and circadian disruption in shift work contributes to excess body weight, and can lead to the identification and optimization of preventative approaches to body weight management in this population.

## 4. Materials and Methods

A total of 24 female day (*n* = 12) and night (*n* = 12) shift workers were recruited and completed participation in this study. Participants were day and night shift working nurses and/or medical staff from New York-Presbyterian Hospital/Columbia University Medical Center, an urban tertiary academic medical center, and worked on a schedule that was either a series of day-oriented work shifts or night-oriented work shifts. Shift durations were either 8 or 12 h. The proportion of individuals working either 8-h shifts (day: 50% vs. night: 33.3%) or 12-h shifts (day: 50% vs. night: 66.6%) did not differ between day and night groups (*p* = 0.41). Participants worked 2 or 3 consecutive shifts before the laboratory visit. Inclusion criteria included female sex, age ≥ 18 y, BMI ≥ 25 kg/m^2^, and consistently working on either a day or night shift schedule. Exclusion criteria included rotating shift schedules, major psychiatric or medical problems (by self-reported health history), travel across time zones within 4 weeks of study, currently being pregnant, currently breastfeeding, or having a child less than 1 year old at home.

After the completion of their respective series of work shifts (i.e., on their first day off), participants attended a laboratory visit to objectively assess food intake. Participants were asked to come to the laboratory within 1 h of awakening from the sleep period following their final work shift. Participants were asked to refrain from consuming any food prior to the laboratory visit.

Body weight, height, and blood pressure were measured upon laboratory entry. Participants then underwent the ad libitum test meal. The test meal consisted of a uniform presentation of foods offered as a buffet. At screening, participants completed a food allergy form to ensure that they could consume all foods that were presented. During the test meal, participants were seated alone and were instructed to eat as much or as little as they want. Participants were given a 30-min eating opportunity, although all participants completed their meal before the maximum time allotment. A range of items, macronutrient and food types, as well as perceived palatability and healthfulness, were presented. The 14-item test meal consisted of 6 major food categories: high fats, high protein, high carbohydrates, sweets/high sugar, fruits, and beverages (Table 1). All food items were weighed before and after the test meal, using a top-loading balance accurate to 0.1 g. Food consumed in the test meal was analyzed using MyFitnessPal, a commercially available web-based nutrition tracking service. Although MyFitnessPal is not a research-grade software, it has been shown to have high agreement with the gold standard Nutrition Data System for Research (NDSR) for assessing energy (*r* = 0.93) and macronutrient content (carbohydrate and protein: *r* = 0.93; fat: 0.73) [33]. Dietary analyses were conducted by individuals with a Masters in Human Nutrition (YC, SL).

The sleep episode preceding the laboratory visit was monitored objectively with the use of wrist-mounted accelerometers (wGT3X-BT Actigraph monitor, ActiGraph LLC, Pensacola, FL, USA). ActiLife 6 data analysis software was used to obtain measures of sleep duration based on Cole-Kripke criteria [34].

The Institutional Review Board of Columbia University Medical Center approved the study procedures, and all participants provided written informed consent.

Primary outcomes were total energy, and macronutrients (fat, protein, and carbohydrate as a percentage of total energy), consumed during the ad libitum test meal. Unpaired samples t-tests were used to compare values between day and night shift groups. Based on these planned comparisons of primary outcomes, we did not apply corrections for multiple comparisons of macronutrient intakes. P-values are based on two-tailed tests. Data are expressed ± standard deviation, unless otherwise indicated. Analyses were conducted using SPSS Statistics for Windows, Version 24.0 (IBM Corp., Armonk, NY, USA).

## Figures and Tables

**Table 1:** Serving size, energy and macronutrient content of food items presented in the ad libitum test meal buffet.

Food Item	Serving Size (g)	Energy (kcal)	Fat (%)	Protein (%)	Carbohydrate (%)
High fat					
Soft cheese	156	483	73	27	0
Peanut butter	188	1190	71	13	16
Oreo cookies	130	668	38	2	60
Chocolate kisses	47	268	29	28	43
High protein					
Greek yogurt	320	298	18	31	51
Protein bar	140	583	29	28	43
High carbohydrate					
Strawberry jam	44	116	0	0	100
Grape jam	41	139	0	0	100
White bread	118	325	9	11	80
Fruits					
Apples	391	203	0	0	100
Bananas	362	301	3	4	93
Raisins	62	204	0	4	96
Beverages					
Apple juice	436	221	0	0	100
Orange juice	441	252	0	2	98

Macronutrient compositions are expressed as percentage of total energy of food item presented.
